# SOX17-mediated LPAR4 expression plays a pivotal role in cardiac development and regeneration after myocardial infarction

**DOI:** 10.1038/s12276-023-01025-w

**Published:** 2023-07-03

**Authors:** Jin-Woo Lee, Choon-Soo Lee, HyunJu Son, Jaewon Lee, Minjun Kang, Jinho Chai, Hyun-Jai Cho, Hyo-Soo Kim

**Affiliations:** 1 Biomedical Research Institute, Seoul National University Hospital, Seoul, Republic of Korea; 2 Department of Molecular Medicine and Biopharmaceutical Sciences, Graduate School of Convergence Science and Technology, and College of Medicine or College of Pharmacy, Seoul National University, Seoul, Republic of Korea; 3 Program in Stem Cell Biology, Seoul National University College of Medicine, Seoul, Republic of Korea; 4 Department of Internal Medicine, Seoul National University Hospital, Seoul, Republic of Korea

**Keywords:** Induced pluripotent stem cells, Bone marrow transplantation, Heart failure, Stem-cell research, Experimental models of disease

## Abstract

Lysophosphatidic acid receptor 4 (LPAR4) exhibits transient expression at the cardiac progenitor stage during pluripotent stem cell (PSC)-derived cardiac differentiation. Using RNA sequencing, promoter analyses, and a loss-of-function study in human PSCs, we discovered that SRY-box transcription factor 17 (SOX17) is an essential upstream factor of LPAR4 during cardiac differentiation. We conducted mouse embryo analyses to further verify our human PSC in vitro findings and confirmed the transient and sequential expression of SOX17 and LPAR4 during in vivo cardiac development. In an adult bone marrow transplantation model using LPAR4 promoter-driven GFP cells, we observed two LPAR4^+^ cell types in the heart following myocardial infarction (MI). Cardiac differentiation potential was shown in heart-resident LPAR4^+^ cells, which are SOX17^+^, but not bone marrow-derived infiltrated LPAR4^+^ cells. Furthermore, we tested various strategies to enhance cardiac repair through the regulation of downstream signals of LPAR4. During the early stages following MI, the downstream inhibition of LPAR4 by a p38 mitogen-activated protein kinase (p38 MAPK) blocker improved cardiac function and reduced fibrotic scarring compared to that observed following LPAR4 stimulation. These findings improve our understanding of heart development and suggest novel therapeutic strategies that enhance repair and regeneration after injury by modulating LPAR4 signaling.

## Heart attack: controlling the signals that repair the heart

Molecular signals that control early heart development could be targeted to repair the heart after a heart attack. Hyun-Jai Cho at Seoul National University Hospital, South Korea, and co-workers previously revealed that lysophosphatidic acid receptor 4 (LPAR4) is expressed widely when embryonic stem cells differentiate into cardiac cells and in adult hearts after a heart attack. Now they have shown that a regulatory protein called SOX17 acts as a crucial mediator of LPAR4. The team identified two types of LPAR4-positive cells in mouse embryos: one type in the heart co-expresses SOX17, while the other in the bone marrow does not. They propose a simple blocker drug that promotes the differentiation of LPAR4 cells in the heart into cardiac cells but prevents inflammation by inhibiting the activity of LPAR4 cells from the bone marrow after a heart attack.

## Introduction

A precise understanding of pluripotent stem cells (PSCs) and cardiac progenitor cells (CPCs) is required for the clinical application of cell therapies targeting the repair and regeneration of the damaged heart^1–3^. In our previous study, we discovered lysophosphatidic acid receptor 4 (LPAR4), a novel cardiac progenitor-specific marker^4^. LPAR4 is a G-protein coupled receptor (GPCR)^5^ transiently expressed in the cardiac progenitor stage during mouse PSC-derived cardiac differentiation and fades out in the mature stage. In an in vivo mouse study, LPAR4^+^ cells were rarely observed in the uninjured adult heart, whereas their number was significantly increased in the infarcted heart. During mouse PSC-derived cardiac differentiation, sequential LPAR4 stimulation using an LPAR4 agonist, followed by blockade of downstream signaling molecules, significantly increased the efficiency of cardiac differentiation. We also showed that cardiac function was improved in a mouse myocardial infarction (MI) model by sequentially stimulating LPAR4 with an agonist and then blocking the downstream signal using a downstream signaling molecule blocker^4^.

Both mouse and human LPAR4 genes and proteins show a homology of over 90%, and the transient expression of human LPAR4 was observed during human-induced pluripotent stem cell (iPSC)-derived cardiac differentiation, as with mouse PSC-derived cardiac differentiation.

In this study, we aimed to investigate the regulatory mechanism of LPAR4 expression during cardiac development and the role of LPAR4 in cardiac repair and regeneration. First, we attempted to identify a specific upstream transcription factor that regulates the transient expression of LPAR4 during the early stages of cardiac differentiation in human PSCs. Second, we generated LPAR4 promoter-driven GFP transgenic mice to trace and characterize LPAR4^+^ cells during in vivo development. Third, we discovered two types of LPAR4^+^ cells in the adult heart after bone marrow transplantation and MI and examined the role of heart-resident LPAR4^+^ cells. Finally, we established the most effective LPAR4 modulation strategy for clinical application to enhance cardiac repair and regeneration after injury in adults.

## Materials and methods

### RNA isolation

Total RNA was isolated using TRIzol reagent (Thermo Fisher Scientific, Inc., Waltham, MA, USA). RNA quality was assessed using the RNA 6000 Nano Chip (Agilent Technologies, Amstelveen, The Netherlands) and an Agilent 2100 bioanalyzer, while RNA quantification was performed using an ND-2000 spectrophotometer (Thermo Fisher Scientific, Inc.).

### Library preparation and whole mRNA sequencing

For control and test RNAs, library construction was performed using the QuantSeq 3′ mRNA-Seq Library Prep Kit (Lexogen, Inc., Vienna, Austria) according to the manufacturer’s instructions. In brief, 500 ng of total RNA was prepared, and an oligo-dT primer containing an Illumina-compatible sequence at its 5′ end was hybridized into the RNA to initiate reverse transcription. After degradation of the RNA template, second strand synthesis was initiated using a random primer containing an Illumina-compatible linker sequence at its 5′ end. The double-stranded library was purified using magnetic beads. Subsequently, the library was amplified to allow the addition of complete adapter sequences required for cluster generation, followed by purification. High-throughput (single-end 75) sequencing was performed using NextSeq 500 (Illumina, Inc., San Diego, CA, USA).

### Data analysis

QuantSeq 3′ mRNA-Seq reads were aligned using Bowtie2 (Langmead and Salzberg, 2012). Bowtie2 indices were either generated from the genome assembly sequence or the representative transcript sequences for alignment to the genome and transcriptome, respectively. The alignment file was used for assembling transcripts, estimating their abundances, and detecting the differential expression of genes. Differentially expressed genes were determined based on counts from unique and multiple alignments using coverage in Bedtools (Quinlan AR, 2010). Read count (RT) data were processed based on the global normalization method using Genowiz ™ version 4.0.5.6 (Ocimum Biosolutions, Telangana, India). Gene classification was based on searches performed using DAVID (http://david.abcc.ncifcrf.gov/) and Medline databases (http://www.ncbi.nlm.nih.gov/).

### Human iPS cell (hiPSC) line

We constructed an hiPSC line by reprogramming newborn foreskin fibroblast cells (NuFF, GSC-3006G; AMS Biotechnology (GlobalStem), Abingdon, UK) through transduction with the four Yamanaka factors. The prepared hiPSC line was cultured on an STO feeder cell (SIM, ATCC® number: CRL-1503™, Manassas, VA, USA).

### Real-time PCR

RNA was isolated and purified from cells harvested at the representative time points using the RNeasy® mini kit (74104, Qiagen, Hilden, Germany) and QIAshredder. A qPCR RT master mix (FSQ-201, TOYOBO, Osaka, Japan) was used to synthesize cDNA from RNA. Primer sequences are shown in Supplementary Table 1 in the Supplementary Information.

### Western blotting

Western blotting was performed to confirm the protein expression patterns of MESP1 and SOX17 during hiPSC-derived cardiac differentiation: anti-MESP1 (sc-130461, Santa Cruz Biotechnology, TX, USA), anti-SOX17 (AF1924, R&D Systems, Minneapolis, MN, USA), and anti-GAPDH (sc-47724, Santa Cruz Biotechnology, TX, USA). Thirty micrograms of protein was transferred onto a nitrocellulose membrane (IB23001, Thermo Fisher Scientific, Inc.) using an iBlot 2 Gel Transfer Device (IB21001, Thermo Fisher Scientific, Inc.). HRP-donkey anti-mouse IgG (H+L) (715-035-151, Jackson ImmunoResearch, Philadelphia, PA, USA) and HRP-donkey anti-goat IgG (H+L) (705-035-147, Jackson ImmunoResearch) were used as secondary antibodies.

### Fluorescence-activated cell sorting (FACS)

We harvested bone marrow (BM) cells from BM, tibias, and femurs and passed them through a 40-μm cell strainer (352340, Corning, New York, USA) to obtain single cells. We also obtained single cells from hearts by dissecting them into the smallest size possible using dissection scissors, incubating them with heart digestion enzymes at 37 °C for 1 h, and passing them through a 70-μm cell strainer (352350, Corning, New York, USA). Isolated single cells from the BM and heart were incubated on ice for 30 min with the following antibodies for flow cytometry analysis: LPAR4 (PA549727, Thermo Fisher Scientific, Inc.), GFP (ab13970, Abcam, Cambridge, UK), CD45 (sc-53665, Santa Cruz Biotechnology), and Nkx2.5 (ab91196, Abcam).

### Immunofluorescence (IF)

Mouse heart and liver tissues were harvested at representative time points relative to MI after bone marrow transplantation (BMT) and embedded in paraffin blocks. Following sectioning (4 μm), IF was performed. Sectioned tissues were incubated with the following antibodies: GFP (ab13970, Abcam), αSA (A2172, Sigma-Aldrich, St. Louis, MO, USA), LPAR4 (PA549727, Thermo Fisher Scientific, Inc.), SOX17 (AF1924, R&D Systems), Sca1 (13-5981-28, Thermo Fisher Scientific, Inc.), and Nkx2.5 (ab91196, Abcam).

### Immunohistochemistry (IHC)

LPAR4_CreERT2_IRES_EGFP_TG mouse embryos were harvested at embryonic Days 10.5 and 12.5. Embryos were fixed in 4% paraformaldehyde solution (163-20145, Wako, Richmond, VA, USA) before being embedded in paraffin. The embryo samples were then sectioned into 4-μm-thick pieces and deparaffinized. Antigens were retrieved by boiling at 95 °C in Tris-EDTA buffer, pH 9.0 (ab93684, Abcam). Next, sections were washed in 1X PBS and blocked in 0.1% BSA with 0.1% Triton X-100 for 1 h. GFP was detected using anti-GFP antibodies (ab13970, Abcam) at 4 °C overnight. Anti-chicken IgY-peroxidase antibodies (A9046, Sigma-Aldrich) were used as secondary antibodies, and the samples were incubated at RT for 90 min. The sections were then developed with the Pierce DAB Substrate kit (34002, Thermo Fisher Scientific, Inc.) at RT for 10 min and counterstained with hematoxylin (03971, Sigma). Images were acquired using a Nikon ECLIPSE Ci-L.

### ChIP assay

For identification of the MESP1 and SOX17 binding sites, the consensus sequences of MESP1 and SOX17 were compared with the sequence located 3017 bp upstream from the LPAR4 transcription start site. For cell crosslinking, 1% formaldehyde (F8775, Sigma-Aldrich) was added to a cell culture dish, fixed at 25 °C for 10 min, and collected in a tube. ChIP RIPA buffer (50 mM Tris-Cl, 1 mM EDTA, 0.5% Triton X-100, 0.1% SDS, 0.1% sodium deoxycholate (SDC), 140 mM NaCl, 1× protease inhibitor cocktail (PIC)) was added to the cell pellet. For DNA shearing, both fractions were sonicated 15 times using a Bioruptor (30 s on/30 s off per cycle). Sonicated DNA was treated with 2 μg each of MESP1 (sc-130461, Santa Cruz Biotechnology, TX, USA) and SOX17 (AF1924, R&D Systems) antibodies overnight. Protein A/G Sepharose (ab193262, Abcam) was added to pull down the antibody-bound DNA, which was then washed three times with wash buffer. DNA was incubated at 65 °C for at least 2 h for decrosslinking. Finally, DNA was recovered using a PCR purification kit (28104, Qiagen) and analyzed using semiquantitative PCR.

### Luciferase assay

HiPSC-derived cells on Day 4 of cardiac differentiation were simultaneously transfected with the firefly pGL3-control luciferase reporter vector (E1751, Promega, Madison, WI, USA) and the *Renilla* luciferase reporter plasmid pRL-TK (E2241, Promega) using the FuGENE® HD transfection reagent (E2311, Promega).

The Dual-Glo® Luciferase Assay system (E2920, Promega) was used to detect LPAR4 promoter-driven luciferase activity according to the manufacturer’s protocol.

### Animals

For animal experiments, 60 C57BL/6 wild-type mice were purchased from Orient Bio (Seongnam-si, Seoul, Korea) and used to generate the murine MI model. Mice were allowed to adapt to the animal facility for 7 days before the experiments. All mice were free of murine viruses, pathogenic bacteria, and endo- as well as ectoparasites. All animal experiments were approved by the Institutional Animal Care and Use Committee (IACUC, IACUC no.: SNU-201028-3-1) of the Seoul National University Hospital.

### Lineage tracing mouse model and TG DNA sample preparation

The DNA prep for the microinjection of the LPAR4_CreERT2_IRES_EGFP TG vector was as follows**:**

The LPAR4_CreERT2_IRES_EGFP plasmid DNA for microinjection comprised a fragment (~6.6 kb) containing the LPAR4 promoter. AfeI and FspI restriction enzymes (each located at the start and outside part of the promoter) were used to secure the fragment. The ~1.9 kb backbone fragment was excised through gel extraction, followed by the introduction of the ~6.6 kb fragment.

### Generation of TG mice

LPAR4_CreERT2_IRES_EGFP_TG mice were generated by Macrogen, Inc. (Seoul, Korea), and they were maintained in a pathogen-free environment. All manipulations were conducted with the approval of the Macrogen Institutional Animal Care and Use Committee (IACUC no.: 2018-01). C57BL/6N female mice were treated with PMSG and hCG for superovulation. PMSG (7.5 IU) and hCG were intraperitoneally (i.p.) injected at 48-h intervals (5 IU) into 5- to 8-week-old female mice. After the hCG injection, female mice were mated with C57BL/6N stud male mice. The next day, following an impregnation check using a vaginal plug, female mice were sacrificed (as a method of euthanasia, 5–6 mg of phenobarbital sodium was i.p. injected), and the fertilized embryos were harvested. LPAR4_CreERT2_IRES_EGFP DNA was comicroinjected into single-cell embryos. Standard microinjection procedures were used for the production of transgenic mice (Macrogen, Inc.). DNA (4 ng/μL) was directly injected into the male pronucleus of the zygote using a micromanipulator, and microinjected embryos were incubated at 37 °C for 1–2 h. In total, 14–16 injected single-cell staged embryos were transplanted by surgical methods into the oviducts of pseudopregnant recipient mice. After F0 was born, genotyping was performed using tail cut samples, and the presence of the transgene was confirmed using PCR analyses of their genomic DNA (forward: 5′-CTGCAAAGACAGGCAGACAAG-3′, reverse: 5′-CATTGCTGTCACTTGGTCGTG-3′).

### Bone marrow transplantation

C57BL/6 wild-type mice were sublethally irradiated (7.5 Gray) using a Cesium-137 Irradiator (IBL437C, CIS Bio, Inc., Codolet, France). Donor mice (LPAR4_CreERT2_IRES_EGFP_TG mice) were euthanized, and GFP^+^ BM cells were collected from the tibia and femur. Next, 5 × 10^7^ GFP^+^ BM cells were systemically transplanted via the jugular vein into sublethally irradiated recipient mice (C57BL/6 wild-type mice). We used a mouse model and performed ligation of the left anterior descending artery after BMT. After 4 weeks of BMT, FACS analysis was performed against GFP^+^CD45^+^ cells to measure the donor engraftment rate.

### Statistical analysis

All data are expressed as the mean ± standard error of the mean (SEM). One-way analysis of variance (ANOVA) using Newman‒Keuls multiple comparison tests was used to compare each group. GraphPad Prism v8 software was used (GraphPad Software, Inc., San Diego, CA, USA), and a *p*-value < 0.01 was considered statistically significant.

## Results

### An upstream regulator of LPAR4 expression during cardiac differentiation in human PSCs

**Fig. 1:**
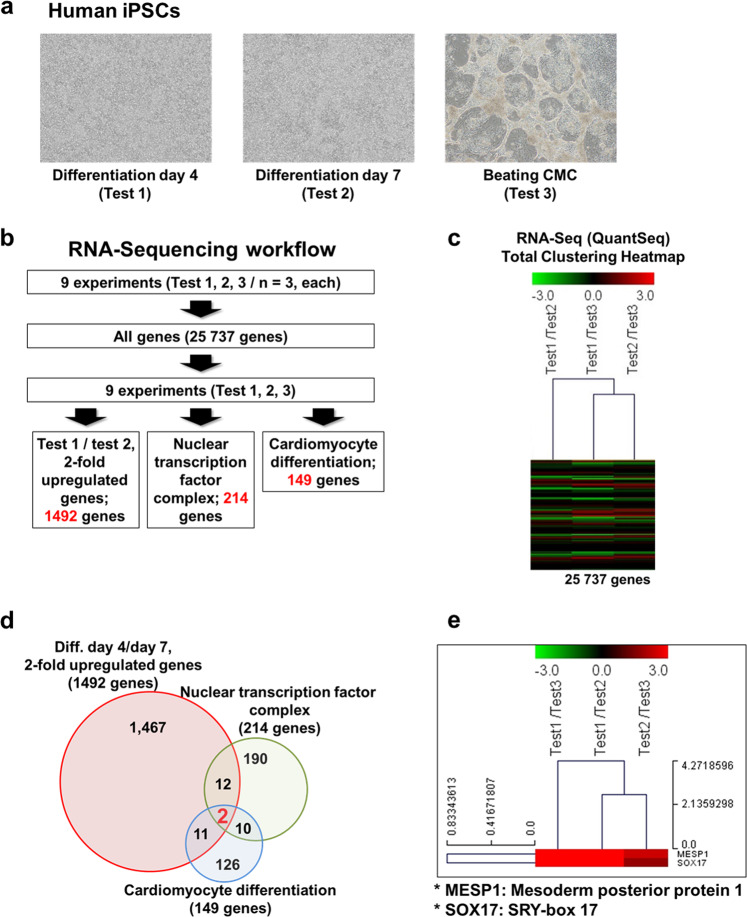

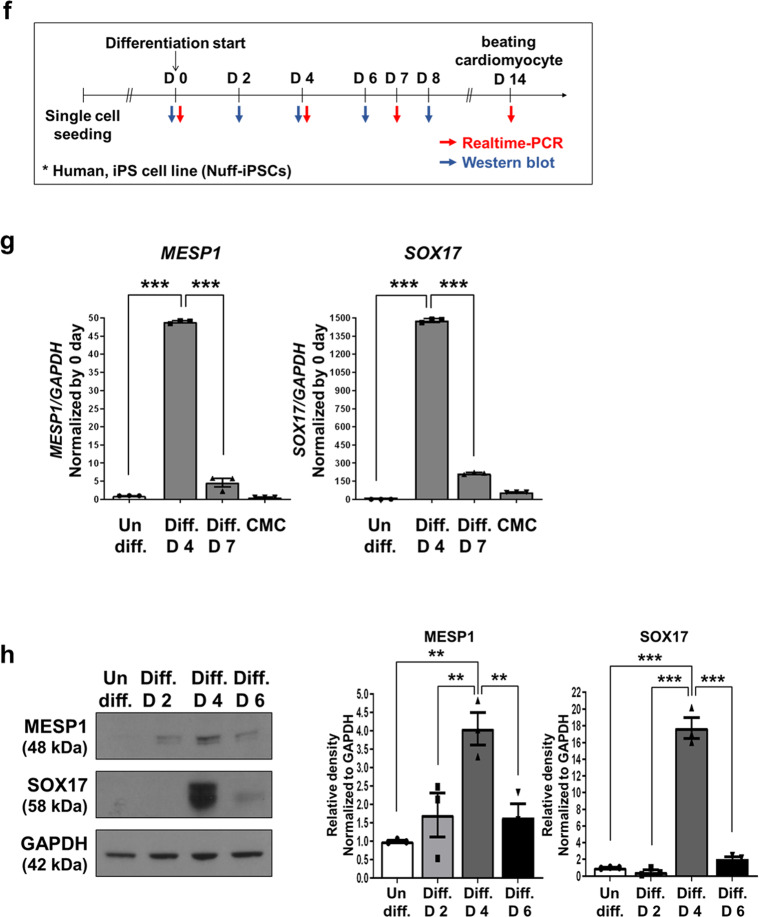
Identification of the LPAR4 upstream transcription factor in hiPSC-derived cardiac differentiation. **a** Brightfield images during hiPSC-derived cardiac differentiation on Days 4 and 7 and in beating cardiomyocytes (CMCs). **b** RNA sequencing workflow for the three cell groups (*n* = 3). **c** Hierarchical cluster analyses of RNA sequencing during cardiac differentiation. **d** Clustering for the identification of LPAR4 upstream transcription factor candidates. **e** A heatmap showing candidate upstream transcription factors of LPAR4. **f** A schematic figure showing harvest days for real-time PCR and western blotting during hiPSC-derived cardiac differentiation. **g** Real-time PCR analysis of the expression of LPAR4 upstream transcription factor candidates during cardiac differentiation. Error bars represent the SEM, ***p < 0.001, unpaired *t*-test (*n* = 3 biological replicates). **h** Left, western blot analysis of LPAR4 expression of upstream transcription factor candidates during cardiac differentiation. Right, quantification of the obtained results (**p < 0.05, ***p < 0.001, means ± SEMs, three technical replicates). All experiments were conducted at least in triplicate.

To identify the LPAR4 upstream transcription factor that regulates the expression of LPAR4, we performed whole mRNA sequencing (RNA sequencing) analysis using samples from three different differentiation points during human-induced pluripotent stem cell (hiPSC)-derived cardiac differentiation. Figure 1a shows (Test 1) hiPSC-derived cardiac differentiation on Day 4, the mesodermal differentiation state, (Test 2) Day 7, the cardiac progenitor state, and (Test 3) beating cardiomyocytes (CMCs). We used a previously described optimized hiPSC-induced cardiac differentiation protocol (Supplementary Fig. 1a)^4^ and obtained hiPSC-derived CMCs on cardiac differentiation Days 14–16. To identify the LPAR4 upstream transcription factor, we selected three clusters from the RNA sequencing results and analyzed 25,737 genes (Fig. 1b, c). Based on the RNA sequencing, we clustered (1) genes that were upregulated more than twofold on Day 4 of hiPSC-derived cardiac differentiation compared with those on Day 7 (1492 genes), (2) nuclear transcription factor complex genes (214 genes), and 3) cardiomyocyte differentiation genes (149 genes) (Fig. 1d). Finally, we selected two candidate transcription factors, mesoderm posterior bHLH transcription factor 1 (MESP1) and SRY-box transcription factor 17 (SOX17), as LPAR4 upstream regulatory factors (Fig. 1e).

To confirm the RNA sequencing results, we then analyzed the RNA and protein expression levels of MESP1 and SOX17 during cardiac differentiation using real-time PCR and western blotting, respectively (Fig. 1f). We observed that the MESP1 and SOX17 expression levels were the highest on Day 4 of human cardiac differentiation (Fig. 1g, h). As their expression was highest at a time point earlier than cardiac differentiation Day 7, when the LPAR4 expression level was the highest, both could be considered candidate LPAR4 upstream transcription factor genes. We verified the expression of LPAR4 and cardiac lineage markers in hiPSC-derived cells during cardiac differentiation using real-time PCR (Supplementary Fig. 1b, c).

### SOX17, but not MESP1, regulates transient LPAR4 expression during cardiac differentiation in human PSCs

**Fig. 2:**
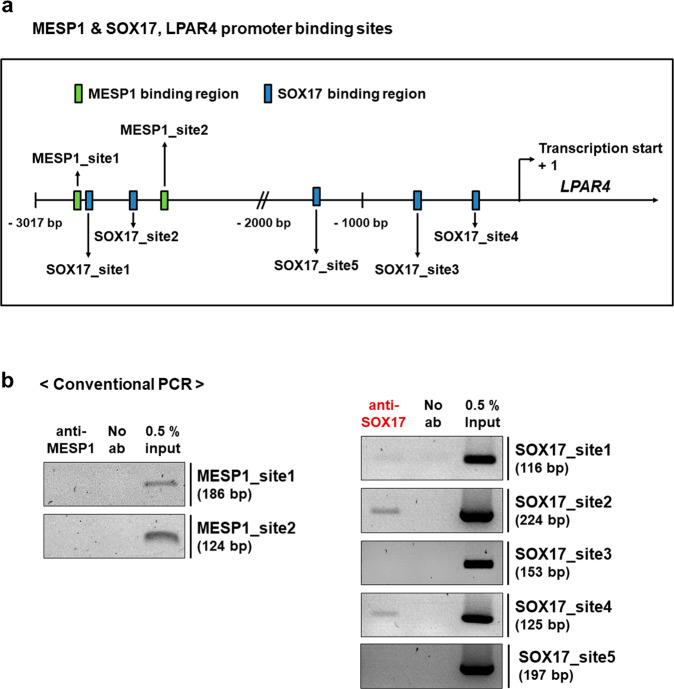
Detection and binding sites of an LPAR4 upstream transcription factor. **a** Schematic showing the human MESP1 and SOX17 binding sites to the human LPAR4 promoter region targeted for chromatin immunoprecipitation (ChIP) assay and mutagenesis. Human LPAR4 promoter region of 3017 bp. **b** Binding of MESP1 or SOX17 to LPAR4 promoter regions using ChIP assays.

**Fig. 3:**
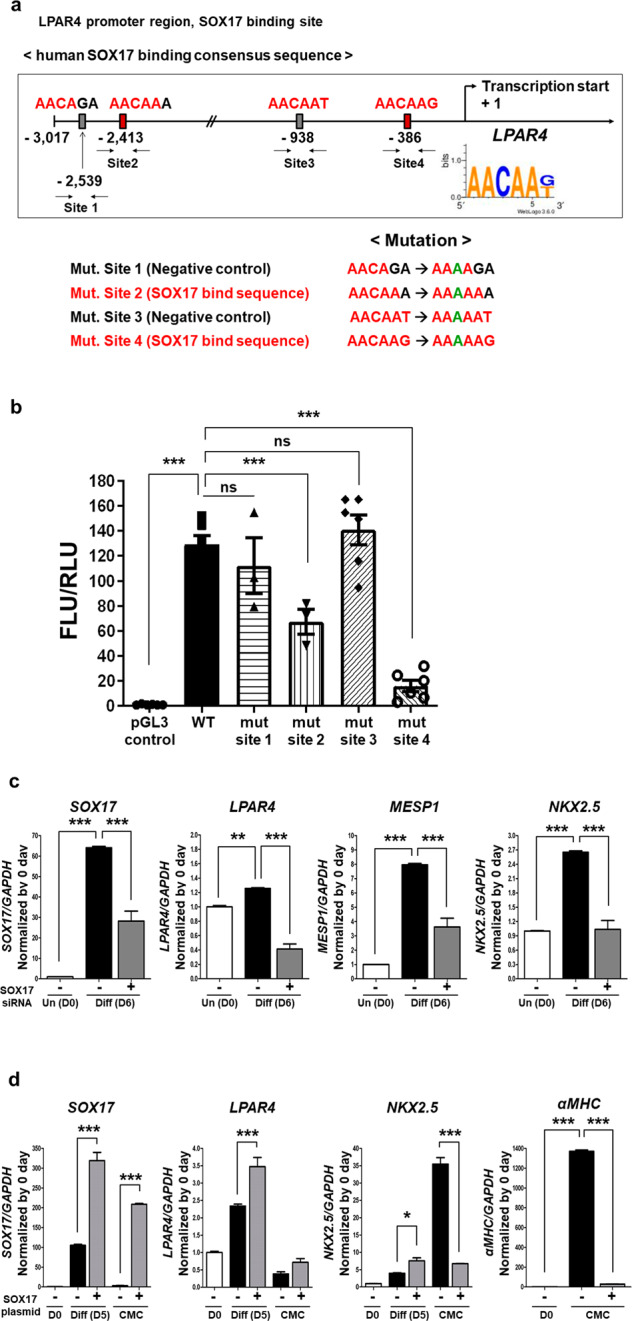
Mutation of the human LPAR4 promoter region to which human SOX17 binds and a gain or loss of function study for SOX17. **a** Schematic figure for SOX17 binding site mutations in the LPAR4 promoter region. A point mutation (C to A) in AACAAG(T), the consensus sequence that binds SOX17, was performed. Based on the ChIP assay results, mutation (mut.) sites 1 and 3 were used as negative controls, and site 5 was excluded because these sites do not bind SOX17, whereas mut. sites 2 and 4 are sites known to bind SOX17. A luciferase assay was performed with these point mutation constructs. **b** Activation of the human LPAR4 gene promoter in hiPSC-derived cells at cardiac differentiation Day 4 using the luciferase assay. ns; not significant, ***p < 0.001. All experiments were conducted at least in triplicate. **c** Real-time PCR analysis of mRNA expression levels of SOX17, LPAR4, and the early cardiac markers MESP1 and Nkx2.5 after cardiac differentiation using SOX17 knockdown (using SOX17 siRNA) hiPSCs. Statistical analyses were performed using one-way ANOVA (Newman‒Keuls). **p < 0.05, ***p < 0.001. **d** Real-time PCR analysis of mRNA expression levels of SOX17, LPAR4, Nkx2.5, and the mature cardiac marker αMHC after cardiac differentiation using SOX17-overexpressing hiPSCs (SOX17 OE). One-way ANOVA (Newman‒Keuls) was performed to assess significant differences (*p < 0.01, ***p < 0.001). All experiments were conducted at least in triplicate.

Next, we aimed to determine which of the two candidate transcription factors (MESP1 and SOX17) binds to the human LPAR4 promoter. We thus examined each consensus sequence capable of binding MESP1 and SOX17 in the LPAR4 promoter region, up to 3017 base pairs (bp) upstream of the transcription start site. We discovered that the LPAR4 promoter region had two (E-box, 5′-CACGTG-3′) and five (5′-AACAAT-3′ or 5′-AACAAAG-3′) binding sites for MESP1 and SOX17, respectively (Fig. 2a). To determine whether MESP1 or SOX17 binds to the LPAR4 promoter region, we performed chromatin immunoprecipitation (ChIP) assay. We observed that MESP1 did not bind to the LPAR4 promoter, whereas SOX17 bound to two of the five sites (Fig. 2b). To further clarify the binding of SOX17 to the LPAR4 promoter region, we first excluded one site that did not bind SOX17 (site 5). Mutagenesis was then performed by changing the third cytosine to adenine in the four SOX17 binding consensus sequences of the LPAR4 promoter region, which was then inserted into a luciferase vector (Fig. 3a) and used to transfect hiPSC-derived cells. We then performed a luciferase assay on hiPSC-derived cells on Day 4 of cardiac differentiation. Consistent with the ChIP assay results, we found that the luciferase activity was maintained at a level similar to that of the wild-type (WT) when SOX17 binding sites 1 and 3 were mutated. In contrast, when SOX17 binding site 2 was mutated, the luciferase activity was reduced to approximately half the intensity of that observed in the WT condition. Furthermore, when SOX17 binding site 4 was mutated, the luciferase activity was reduced to levels similar to those observed in cells transfected with the mock vector (Fig. 3b). Thus, we confirmed that SOX17 is an LPAR4 upstream transcription factor and that it binds to the LPAR4 promoter at two or more sites.

Next, we performed loss-of-function (LOF) and gain-of-function (GOF) studies to verify whether SOX17 expression affects the cardiac differentiation of hiPSCs. We discovered that siRNA-induced SOX17 knockdown inhibited LPAR4 expression as well as cardiac differentiation by reducing the expression of early cardiac lineage markers (Fig. 3c). Interestingly, SOX17 overexpression increased LPAR4 expression while inhibiting cardiac differentiation, thus suggesting that transient upregulation of SOX17 and LPAR4 in the early stage of cardiac development and subsequent fading-out in the later stage is necessary to complete cardiac differentiation (Fig. 3d).

### LPAR4 lineage tracing in vivo during embryonic and postnatal development

**Fig. 4:**
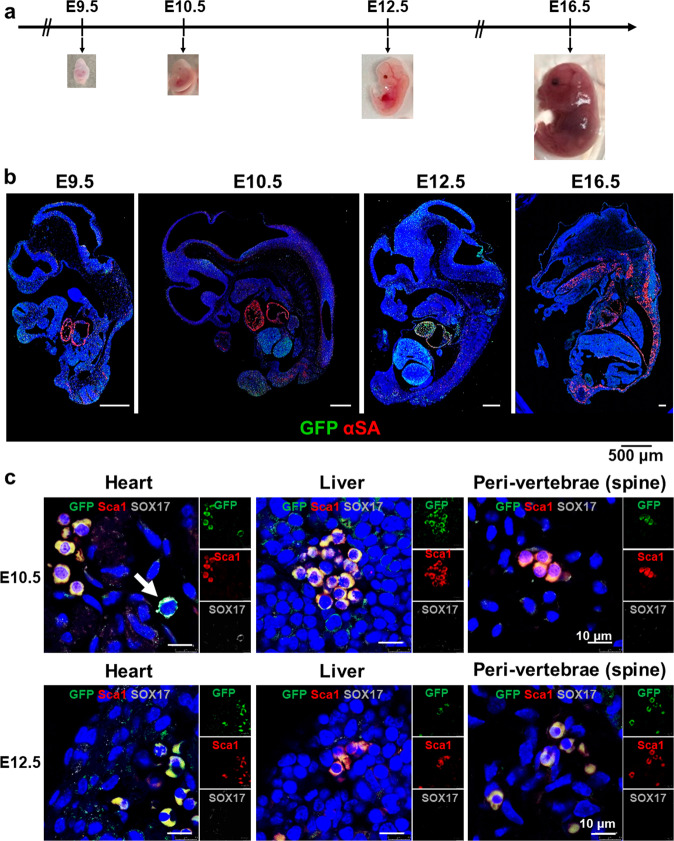

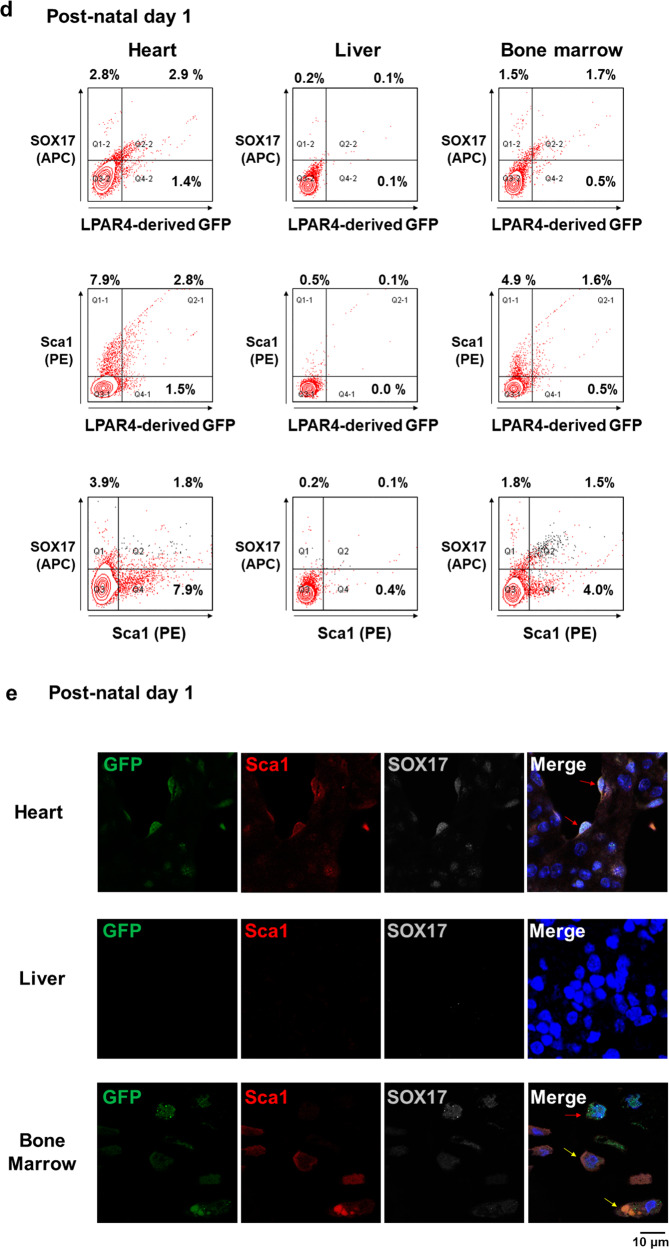
LPAR4 expression during mouse embryonic and postnatal development. **a** Schematic representation of LPAR4 lineage tracing mouse embryo harvesting (embryonic Day 9.5 (E9.5), E10.5, E12.5, and E16.5). At least three mouse embryos were harvested for each time point. **b** Immunostaining for GFP and α-SA expression during LPAR4 lineage tracing mouse embryonic development. Merged images for GFP (green) and α-SA (red) from whole mouse embryos (E9.5, E10.5, E12.5, and E16.5). Green, GFP; red, αSA; blue, DAPI (nuclei). Scale bar, 500 μm. **c** Immunofluorescence analysis of embryonic heart, liver, and BM from LPAR4 lineage tracing mice at E10.5 and E12.5. Green, GFP; red, Sca1; white, SOX17; blue, DAPI (nuclei). Scale bar, 10 μm. All experiments were conducted at least in triplicate. **d** FACS analysis of heart, liver, and BM cells from LPAR4 lineage tracing mouse neonates (postnatal day 1). FACS analysis was performed using GFP (FITC) for LPAR4 expression and SOX17 (APC) or Sca1 (PE) staining. **e** Immunofluorescence analysis of LPAR4 lineage tracing for mouse neonatal heart, liver, and bone marrow cells. Green, GFP; red, Sca1; white, SOX17, blue, DAPI (nuclei).

Next, we performed in vivo mouse embryo analyses to confirm the hiPSC in vitro cardiac differentiation findings. We constructed an LPAR4 promoter-driven GFP transgenic mouse line to trace the LPAR4 lineage and verify the expression of LPAR4 during in vivo embryonic development (Supplementary Fig. 2)^4^. We harvested embryos on embryonic Days 9.5 (E9.5), 10.5 (E10.5), 12.5 (E12.5), and 16.5 (E16.5) (Fig. 4a).

To confirm the expression of GFP under LPAR4 promoter activation in mice, we performed double-stained IF analysis of GFP and the heart-specific marker α-sarcomeric actinin (α-SA) on harvested embryos (Fig. 4b). Following a comparison of the hearts of embryos from different developmental stages, we observed GFP expression differences between E10.5 and E12.5. As the expression of LPAR4-derived GFP was the strongest in the E12.5 hearts, we expected the expression of the upstream regulator SOX17 to be mainly detected at E10.5. The presence of SOX17 and GFP double-positive cells was observed at E10.5 but not at E12.5 (Fig. 4c). Interestingly, we observed LPAR4 expression at E10.5 and E12.5 in the liver and peri-vertebrae (spine); however, no SOX17 and LPAR4 double-positive cells were detected (Fig. 4c). To further analyze LPAR4 expression in embryonic heart, liver, and bone marrow cells, we performed immunohistochemistry (IHC) on E10.5 and E12.5 embryos (Supplementary Fig. 3a, b). As expected, embryonic hearts showed strong GFP expression, which increased from E10.5 to E12.5. However, in the embryonic liver and spine (peri-vertebrae), the overall expression of GFP was weak. Combining IF and IHC data, we concluded that a small number of cells in the embryonic liver and bone marrow strongly express LPAR4. Finally, these data indicate that two types of LPAR4^+^ cells are present in vivo and that SOX17-mediated LPAR4 expression is specific for cardiac development.

We also assessed the presence of stem cell antigen-1 (Sca1)^6,7^, which is both a hematopoietic and cardiac progenitor cell marker, in mouse embryos using fluorescence-activated cell sorting (FACS) and immunofluorescence (IF) analyses. We discovered that both cardiac and hematopoietic lineage cells were LPAR4 and Sca1 double-positive during the embryonic (Fig. 4c) and neonatal stages (Fig. 4d, e). Hematopoietic cells were observed in the embryonic liver and peri-vertebrae (Fig. 4c). Sca1 and LPAR4 double-positive cells were almost nonexistent in the neonatal liver but were common in the neonatal bone marrow (BM), thus suggesting the migration of hematopoietic cells from the embryonic liver to neonatal BM (Fig. 4d). SOX17 and LPAR4 double-positive cells, on the other hand, were scarcely found in the neonatal BM with a low expression level of SOX17 (Fig. 4e). Moreover, LPAR4/Sca1/SOX17 triple-positive cells were only present in the neonatal heart and not in the liver or BM (Fig. 4e).

Overall, we confirmed the transient and sequential expression of SOX17 and LPAR4 during in vivo cardiac development and identified two types of LPAR4^+^ cells. Furthermore, we showed that SOX17 is an important indicator that distinguishes the two types of LPAR4^+^ cells. Cardiac lineage LPAR4^+^ cells express SOX17 transiently during the early embryologic stage, while hematopoietic LPAR4^+^ cells rarely express SOX17 and migrate from the liver to BM.

### Heart-resident and BM-derived LPAR4 cells in the adult mouse heart after injury

**Fig. 5:**
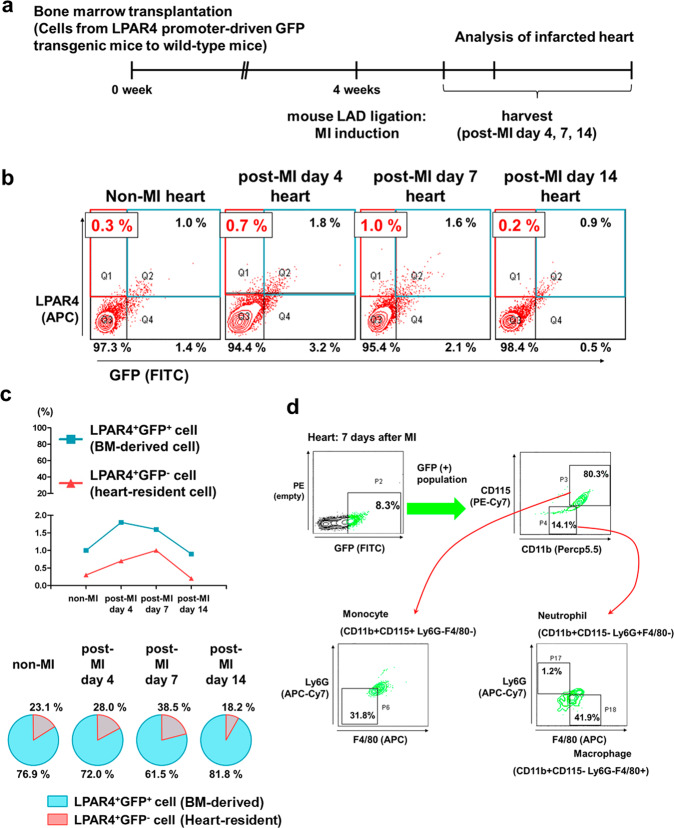

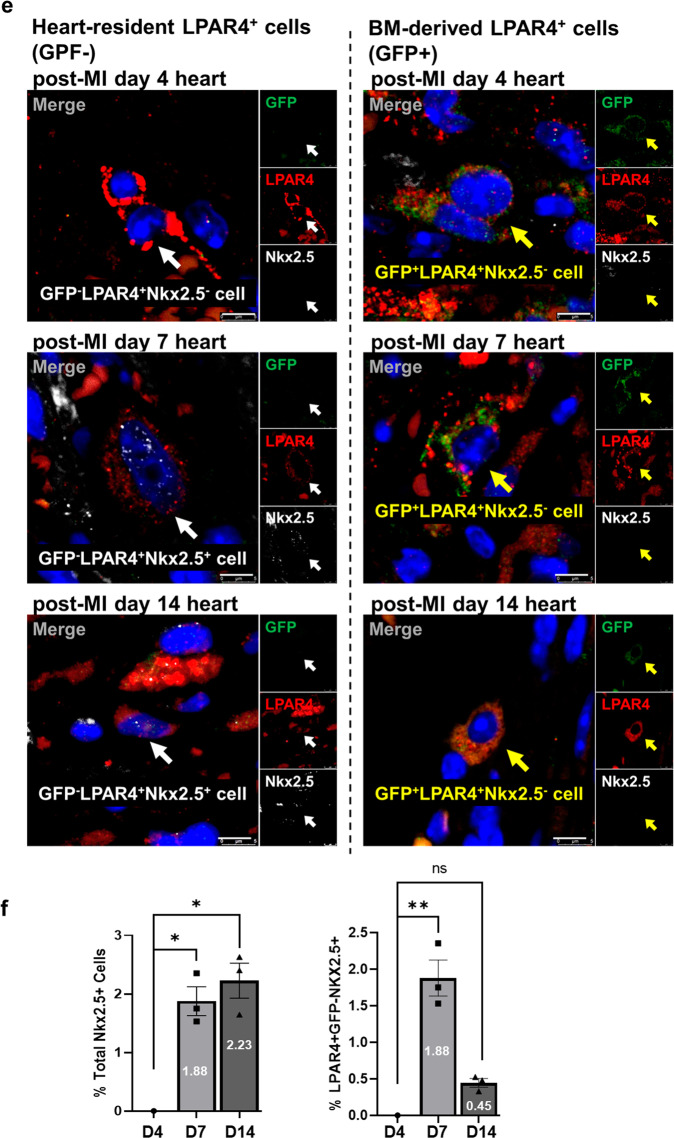
Characterization of LPAR4 in the myocardial infarcted heart after bone marrow transplantation. **a** Schematic figure of mouse bone marrow transplantation (BMT) and the myocardial infarction (MI) model. **b** FACS analysis of LPAR4 and GFP staining in non-MI hearts after BMT and in hearts on Days 4, 7, and 14 post-MI after BMT. **c** Top, line graph showing the percentage of LPAR4^+^GFP^+^ and LPAR4^+^GFP^−^ cells in non-MI and post-MI hearts. Bottom, Venn diagram showing the ratio of LPAR4^+^GFP^+^ and LPAR4^+^GFP^−^ cells in non-MI and post-MI hearts. **d** Representative flow cytometry plots of GFP-positive cells in the heart on Day 7 post-MI after BMT. Monocyte expression (CD11b^+^CD115^+^Ly6G^−^F4/80^−^), macrophage (CD11b^+^CD115^−^Ly6G^−^F4/80^+^), and neutrophil (CD11b^+^CD115^−^Ly6G^+^F4/80^−^) markers. **e** IF analyses were performed on hearts 4, 7, and 14 days after MI in BMT mice. BM-derived cells were stained with GFP (green), heart-resident LPAR4^+^ cells were stained with LPAR4 (red), and cardiac progenitor cells were stained with Nkx2.5 (white). Scale bar, 5 μm. **f** Quantification of the total percentage of Nkx2.5^+^ cells and LPAR4/Nkx2.5 double-positive cells in post-MI hearts. All experiments were conducted at least in triplicate.

A bone marrow transplantation (BMT)^8,9^ and myocardial injury experiment was designed to examine the dynamics and characteristics of heart-resident LPAR4^+^ cells compared with those of BM-derived LPAR4^+^ cells. We harvested BM mononuclear cells from LPAR4 promoter-driven GFP transgenic mice. Donor BM cells were inoculated into wild-type recipient mice through the jugular vein after ablating the cells through 7.5 Gray irradiation. Four weeks later, donor cell engraftment was evaluated using flow cytometry analysis of the recipient BM-repopulated cells (Supplementary Fig. 4). We induced MI by ligating the left anterior descending artery (Fig. 5a, experimental scheme).

We first analyzed non-MI sham hearts and hearts that were 4, 7, and 14 days post-MI using flow cytometry^10^. To identify the number of BM-derived cells that infiltrated into the heart after MI, we counted CD45^+^ cells, a pan leukocyte marker-expressing cell type^11^. CD45^+^ cells rapidly increased at Day 4 post-MI and decreased as MI progressed (Supplementary Fig. 5a, b). Second, we measured the number of heart-resident and BM-derived LPAR4^+^ cells using LPAR4 and GFP double staining, respectively. We observed that the total number of LPAR4-expressing cells progressively increased to Day 4 post-MI, peaked during Day 7, and then gradually decreased (Fig. 5b). We observed the different dynamics of the two types of LPAR4^+^ cells. BM-derived LPAR4^+^ cells were the most prevalent on Day 4 after MI. Heart-resident LPAR4^+^ cells were most numerous on Day 7 post-MI. Thereafter, the number of both types of cells declined (Fig. 5c).

To further characterize BM-derived LPAR4^+^ cells, we analyzed the GFP^+^ population in the heart 7 days after MI (Fig. 5d). Most cells expressed CD11b and CD115, indicating that they were neither T nor B cells. In this population, we rarely observed neutrophils (CD11b^+^CD115^−^Ly6G^+^F4/80^−^). These results therefore suggest that most BM-derived LPAR4^+^ cells that infiltrated the heart following MI were inflammatory cells with characteristics resembling monocytes or macrophages.

Next, we examined post-MI heart tissue using triple-color immunofluorescence staining to confirm the cardiac differentiation potentials of heart-resident versus BM-derived LPAR4^+^ cells (Fig. 5e). We did not detect the expression of Nkx2.5, a cardiac transcription factor and progenitor marker, in BM-derived GFP^+^LPAR4^+^ cells; however, we observed nuclear Nkx2.5 expression in heart-resident GFP^−^LPAR4^+^ cells from post-MI hearts. Nkx2.5-expressing cells started to appear on Day 7 post-MI and then continued to increase; however, the percentage of LPAR4^+^/Nkx2.5^+^ double-positive cells peaked on Day 7 and then decreased to 0.45%, which is similar to the LPAR4 expression pattern in post-MI hearts (Fig. 5f). Furthermore, flow cytometry analysis provided consistent results, indicating that Nkx2.5 was not expressed in BM-derived, heart-infiltrating GFP^+^ cells (Supplementary Fig. 5c).

### The modulation of LPAR4 downstream signaling is critical for cardiac differentiation and the repair process after myocardial infarction

As we discovered that heart-resident LPAR4^+^ cells undergo cardiac differentiation, we designed an experiment to increase the cardiac differentiation and repair efficiency of heart-resident LPAR4^+^ cells after MI. In a previous study, we identified a specific protocol to enhance cardiac differentiation efficiency in vitro and improve cardiac function in vivo through the sequential stimulation and inhibition of LPAR4, respectively.

**Fig. 6:**
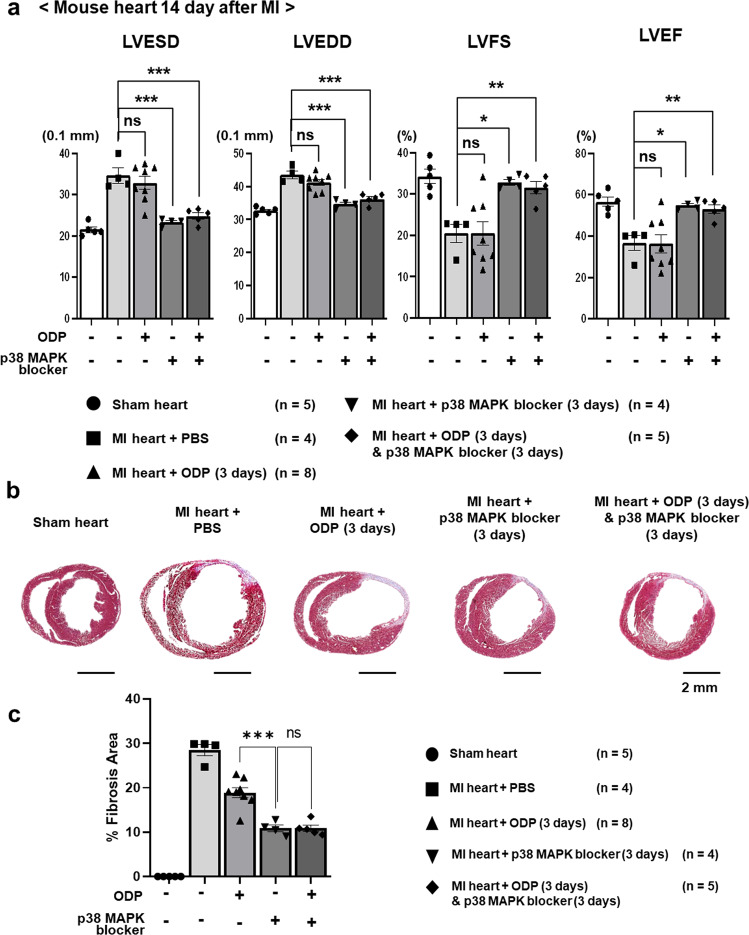
Effect of treatment with a p38 MAPK blocker on cardiac differentiation and heart repair processes. **a** Echocardiography (sham heart group, *n* = 5; MI + PBS group, *n* = 4; MI + octadecenyl phosphate (ODP) [3 d] group, *n* = 8; MI + p38 MAPK blocker (SB203580) [3 d] group, *n* = 4; MI + ODP [3 d] + p38 MAPK blocker [3 d] group, *n* = 5). LVESD left ventricular end-systolic diameter, LVEDD left ventricular end-diastolic diameter, LVFS left ventricular functional shortening, LVEF left ventricular ejection fraction. **b** Hearts were fixed, sectioned, and MT stained. **c** Quantification of percent fibrotic area of MI hearts, analyzed by ImageJ. Statistical analyses were performed using one-way ANOVA (Newman‒Keuls). ns not significant, *p < 0.01, **p < 0.05, ***p < 0.001. Scale bar, 2 mm. All experiments were conducted at least in triplicate.

In this study, we aimed to develop a more effective and efficient protocol. We compared the cardiac function of three experimental groups using an in vivo mouse MI model: LPAR4 stimulation for 3 days immediately after MI, delayed LPAR4 inhibition from post-MI days 3–6 using an LPAR4 downstream signaling molecule, p38 mitogen-activated protein kinase (p38 MAPK) inhibitor, and LPAR4 stimulation for 3 days immediately after MI followed by LPAR4 inhibition for 3 days. The LPAR4 stimulator octadecenyl phosphate (ODP) and inhibitor p38 MAPK blocker were injected subcutaneously. Echocardiographic examination indicated that sequential LPAR4 inhibition after stimulation or delayed LPAR4 inhibition significantly improved cardiac function compared to that observed following the early LPAR4 stimulation strategy (Fig. 6a). When we analyzed the scar in each group using Masson’s trichrome (MT) staining, we observed a reduction in the fibrotic area and alleviated heart wall thinning following sequential LPAR4 stimulation/inhibition or delayed LPAR4 inhibition groups, consistent with the echocardiography results (Fig. 6b, c). Based on these findings, we concluded that the delayed inhibition of LPAR4, but not LPAR4 stimulation, using a specific downstream signaling blocker improved cardiac repair and restored cardiac function during the early stages after MI.

## Discussion

In this study, we observed several novel findings related to the regulatory mechanism of LPAR4 in vitro and in vivo and its potential clinical implication. First, we identified SOX17, an upstream transcription factor that induces the expression of LPAR4 during early cardiac differentiation. Second, we identified two types of LPAR4^+^ cells in the infarcted heart and delineated the fate of heart-resident LPAR4^+^ cells using an LPAR4 lineage tracing mouse model. Third, we suggested an efficient cardiac repair protocol using the molecular blockade of LPAR4 downstream signaling.

In a previous study^4^, we identified a transient expression pattern of LPAR4, in which its expression was increased until the cardiac progenitor stage was reached during cardiac differentiation and then rapidly decreased at the cardiac maturation stage. The expression of LPAR4 is essential for cardiac differentiation to proceed, and its suppression during the early differentiation stages inhibits cardiac maturation. The transcription factors that regulate the expression of LPAR4 during cardiac differentiation were therefore investigated. Using RNA sequencing, ChIP assays, and mutagenesis analyses, we identified SOX17 as the main LPAR4 upstream transcription factor and assessed the importance of SOX17-induced LPAR4 activation during the early stages of cardiac differentiation using an LPAR4 lineage tracing mouse model. We further found that SOX17 exhibits a transient expression pattern similar to that of LPAR4 during cardiac differentiation.

SOX17 is a member of the SOXF subgroup, which is involved in vascular development, and as such, SOX17 complete knockout (SOX17^−/−^) mice show embryonic lethality^12^. Moreover, SOX17 is a representative endoderm marker^13^ and a well-known transcription factor essential for endothelial differentiation^12,14^, and it is also expressed in the early stages of cardiac lineage differentiation^15,16^. Our study revealed that SOX17 induces the expression of LPAR4, which is turned off with differentiation into the cardiac lineage. In addition, we confirmed through IF analysis that cells expressing SOX17 will subsequently differentiate into the cardiac lineage instead of the cells surrounding cardiac lineage cells. We further confirmed through SOX17 overexpression experiments that cardiac differentiation does not proceed further unless SOX17 expression is halted. A decrease in SOX17 expression is expected to suppress the expression of LPAR4; however, the detailed mechanism of action remains to be elucidated.

Next, we investigated the identity of LPAR4^+^ cells by creating an in vivo LPAR4 lineage tracing mouse model. Using SOX17, GFP, and LPAR4 triple staining, we detected the presence of SOX17^+^LPAR4^+^ cells, which were predicted to be heart-resident cells in E10.5 hearts. However, we did not detect any SOX17^+^ cells in E12.5 hearts. These in vivo findings confirm that the LPAR4 upstream transcription factor (SOX17) was initially expressed and then suppressed at an earlier time point compared to the LPAR4 expression patterns observed during mouse embryonic development. In contrast to our previous studies, LPAR4 was strongly expressed in both the heart and certain liver cells during embryonic development in the LPAR4 lineage tracing mouse model.

We also discovered that two types of LPAR4^+^ cells exist in mouse embryos. The main difference between the two types of LPAR4^+^ cells is the transient expression of SOX17. We demonstrated that LPAR4^+^ cells that transiently expressed SOX17 were subsequently becoming cardiac cells that were continuously present in the heart, while the LPAR4^+^ cells that do not express SOX17 were hematopoietic lineage cells that migrated from the liver to the BM; these were identified using Sca1 expression tracing.

After detecting two types of LPAR4^+^ cells in mouse embryos, we designed experiments to determine whether both types of LPAR4^+^ cells were also present in adult mice. As the liver-to-BM migration of Sca1-expressing LPAR4^+^ cells was confirmed through mouse embryo experiments, we hypothesized that Sca1-expressing LPAR4^+^ cells would be present in the adult mouse BM. As few LPAR4^+^ cells were observed in the normal adult mouse hearts, we analyzed adult mouse hearts with MI after BMT and detected both types of LPAR4^+^ cells immediately after MI, similar to that in embryonic mouse hearts. Infiltration of BM cells into the heart following MI has been well documented^17–19^. Although there were few LPAR4^+^ cells in normal hearts (non-MI hearts), the number of LPAR4^+^ cells significantly increased immediately after MI, with FACS analysis showing a more than twofold increase. Immediately after MI, the number of BM-derived LPAR4^+^ cells in the heart was significantly increased, confirming that the number of BM-derived LPAR4^+^ cells infiltrating the heart was more than twofold higher than the number of heart-resident LPAR4^+^ cells. Additionally, the number of CD45^+^ BM-derived cells infiltrating the heart was more than fourfold that of the total number of heart-resident LPAR4^+^ cells. Heart-resident LPAR4^+^ cells and BM-derived LPAR4^+^ cells showed a decreasing trend as MI progressed. An IF assay was performed to investigate the expression of Nkx2.5^20^ and determine the cardiac lineage differentiation capacity of both types of LPAR4^+^ cells, and only heart-resident LPAR4^+^ cells expressed Nkx2.5.

Since LPAR4 plays a critical role in heart development during mouse embryogenesis, there is a high chance that the complete knockout of LPAR4 may elicit embryonic lethality. Therefore, to further investigate the characteristics and in vivo importance of the two LPAR4^+^ cells, we generated two separate inducible and conditional knockout (icKO) mouse models: bone marrow-specific and heart-specific LPAR4 knockouts.

We demonstrated that SOX17 is an upstream regulator of LPAR4. Moreover, by identifying the role of the LPAR4^+^ cell cardiac lineage residing in the heart, we established an efficient cardiac regeneration method applicable to MI heart cases in clinical settings. Although a method for inhibiting LPAR4 after stimulation was established previously, our method is more efficient for improving cardiac function by eliminating the LPAR4 stimulation step and only blocking the LPAR4 downstream signaling molecules. Using this method, we confirmed that LPAR4 inhibition during the late cardiac differentiation phase is more critical for cardiac maturation than LPAR4 stimulation during the early cardiac differentiation phase. The p38 MAPK^21^ signaling pathway in cardiomyocytes has been described in previous studies^22^, and the enhancement of MI cardiac function through p38 MAPK inhibition was also established^23^. Several p38 MAPK blocker drugs are being developed under clinical trials. Losmapimod reached phase III clinical trials in hospitalized MI patients^24^, and acumapimod completed phase II trials in chronic obstructive pulmonary disease (COPD) patients^25^. Therefore, for designing and developing MI therapeutic drugs, we have established a simpler and advantageous protocol using only p38 MAPK blockers.

p38 MAPK is known to have both anti-inflammatory and antifibrotic effects^26,27^. In post-MI hearts, various inflammatory stimuli become activated, which in turn activates p38 MAPK, resulting in fibrotic formation^26^. The p38 MAPK blocker thus inhibits further inflammation events, reducing fibrosis, and induces myofibroblast differentiation^27^. In conclusion, p38 MAPK blockers promote cardiac regeneration by inhibiting inflammation, myofibroblast differentiation, and LPAR4 downstream signaling, thus promoting the cardiac differentiation of heart-resident LPAR4^+^ cells but not BM-derived LPAR4^+^ cells.

In summary, our findings improve the general understanding of heart development and suggest a new therapeutic strategy to enhance repair and regeneration after injury through LPAR4 signaling modulation.

## Supplementary information

Supplemental Material
