# Supplementary material for: SOX17-mediated LPAR4 expression plays a pivotal role in cardiac development and regeneration after myocardial infarction
Source: Exp Mol Med. 2023 Jul 3;55(7):1424–36. doi: 10.1038/s12276-023-01025-w (PMC10394006; doi:10.1038/s12276-023-01025-w)
Supplement: Supplementary file 1 — Supplemental Material [file 12276_2023_1025_MOESM1_ESM.pdf]

Supplemental information

SOX17-mediated LPAR4 expression plays a pivotal role in cardiac development and regeneration after myocardial infarction

Jin-Woo Lee[1,4†], Choon-Soo Lee[1†], HyunJu Son[4†], Jaewon Lee[1], Minjun Kang[4], Jinho Chai[3], Hyun-Jai Cho[2*], and Hyo-Soo Kim[1,2,4]

[1]Biomedical Research Institute, Seoul National University Hospital, Seoul, Republic of Korea; [2]Department of Internal Medicine, Seoul National University Hospital, Seoul, Republic of Korea; [3]Program in Stem Cell Biology, Seoul National University College of Medicine, Seoul, Republic of Korea; [4]Department of Molecular Medicine and Biopharmaceutical Sciences, Graduate School of Convergence Science and Technology, and College of Medicine or College of Pharmacy, Seoul National University, Seoul, Republic of Korea

[†] These authors contributed equally to this article.

Corresponding authors:

*Hyun-Jai Cho, MD, PhD

Professor, Division of Cardiology, Department of Internal Medicine

Seoul National University Hospital, 101 Daehak-ro, Jongno-gu, Seoul 03080, Korea

Tel: (82)-2-2072-3931; Fax: (82)-2-3675-0805

E-mail: hyunjaicho@snu.ac.kr ; hyunjaicho@gmail.com

Supplementary Table 1 – Primers used for PCR

Target gene	Sequence
human GAPDH forward	5′-gaccccttcattgacctcaac-3′
human GAPDH reverse	5′-cttctccatggtggtgaaga-3′
human Mesp1 forward	5′-agcccaagtgacaagggacaact-3′
human Mesp1 reverse	5′-aaggaaccacttcgaaggtgctga-3′
human SOX17 forward	5′-cagaatccagacctgcacaa-3′
human SOX17 reverse	5′-gcggccgctacttgtagtt-3′
human LPAR4 forward	5′-cttgttgggggaagaacaaa-3′
human LPAR4 reverse	5′-ccacctgtttcctggtcact-3′
human T forward	5′-tgtcccaggtggcttacagatgaa-3′
human T reverse	5′-ggtgtgccaaagttgccaatacac-3′
human NKX2.5 forward	5′-tttgcattcactcctgcggagaccta-3′
human NKX2.5 reverse	5′-actcattgcacgctgcataatcgc-3′
human cTnT forward	5′-gtgggaagaggcagactgag-3′
human cTnT reverse	5′-atagatgctctgccacagc-3′
human αMHC forward	5′-tcagctggaggccaaagtaaagga-3′
human αMHC reverse	5′-ttcttgagctctgagcactcgtct-3′

SUPPLEMENTARY FIGURES

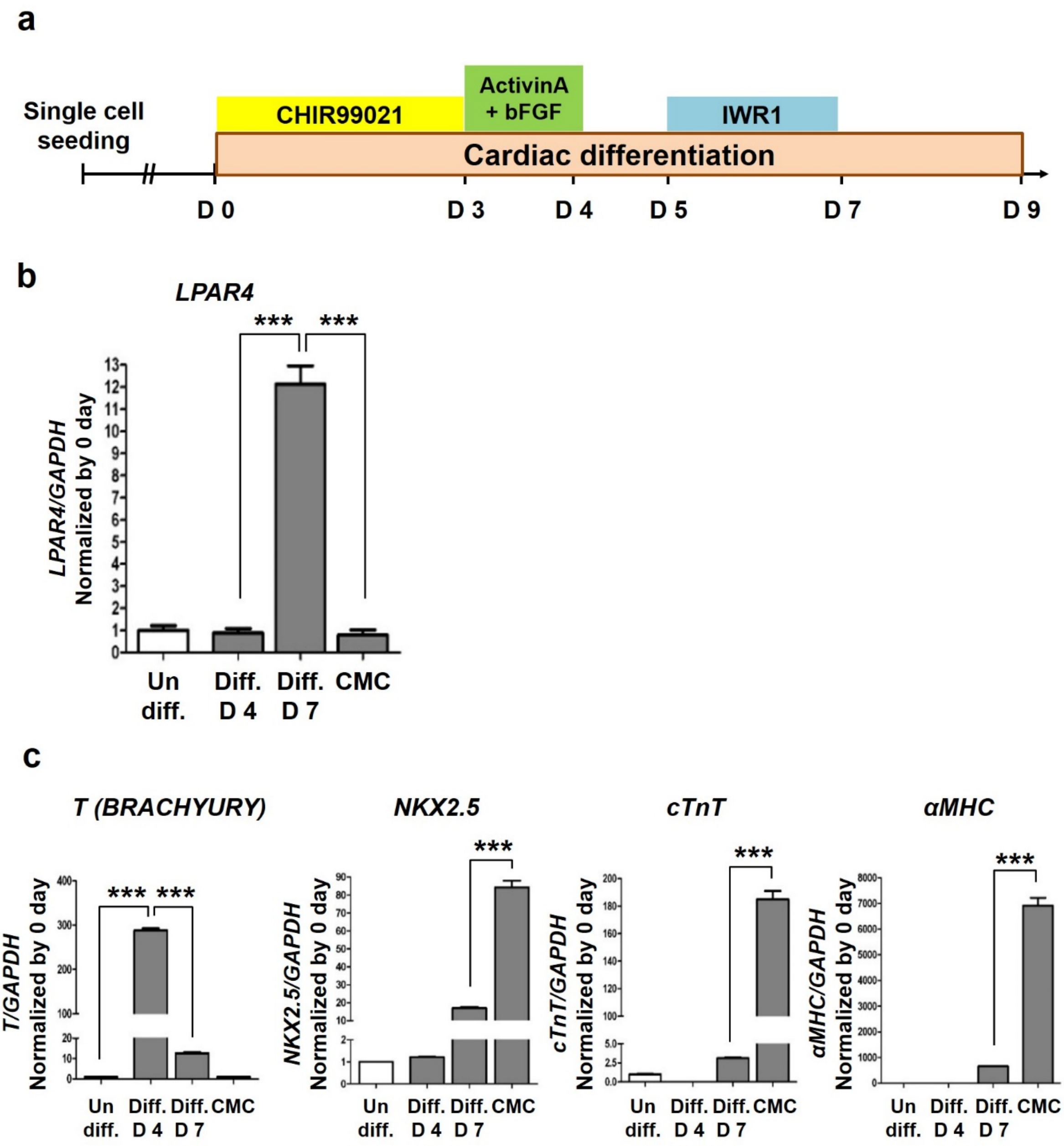

Supplementary Fig. 1. Human iPSC-derived cardiac differentiation protocols and cardiac-related gene expression patterns.

a Schematic representation of the established hiPSC-derived cardiac differentiation protocol. **b** Transient expression of LPAR4 during human cardiac differentiation. Error bars represent SEM; ***p < 0.001, unpaired *t*-test; n = 3 biological replicates. **c** Expression patterns of cardiac-related genes during human cardiac differentiation. Error bars represent SEM; ***p <

0.001, unpaired *t*-test; n = 3 biological replicates. All experiments were conducted at least in triplicate.

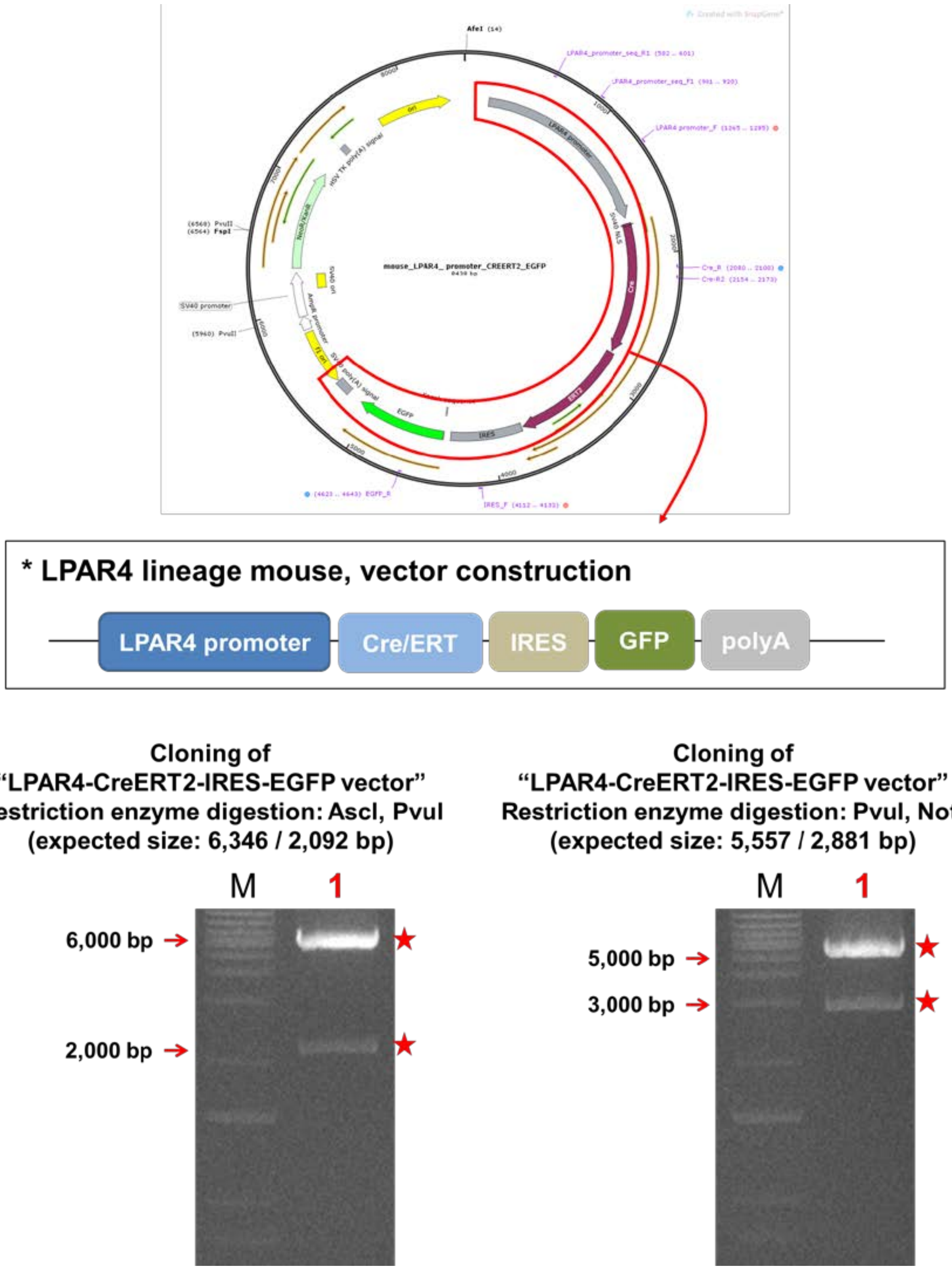

Supplementary Fig. 2. Construction and validation of LPAR4 lineage tracing mouse model vector.

Top, schematic representation of the LPAR4 lineage tracing mouse model vector. Bottom, The cloned LPAR4 lineage tracing vector was cut using restriction enzymes and verified using DNA gel electrophoresis.

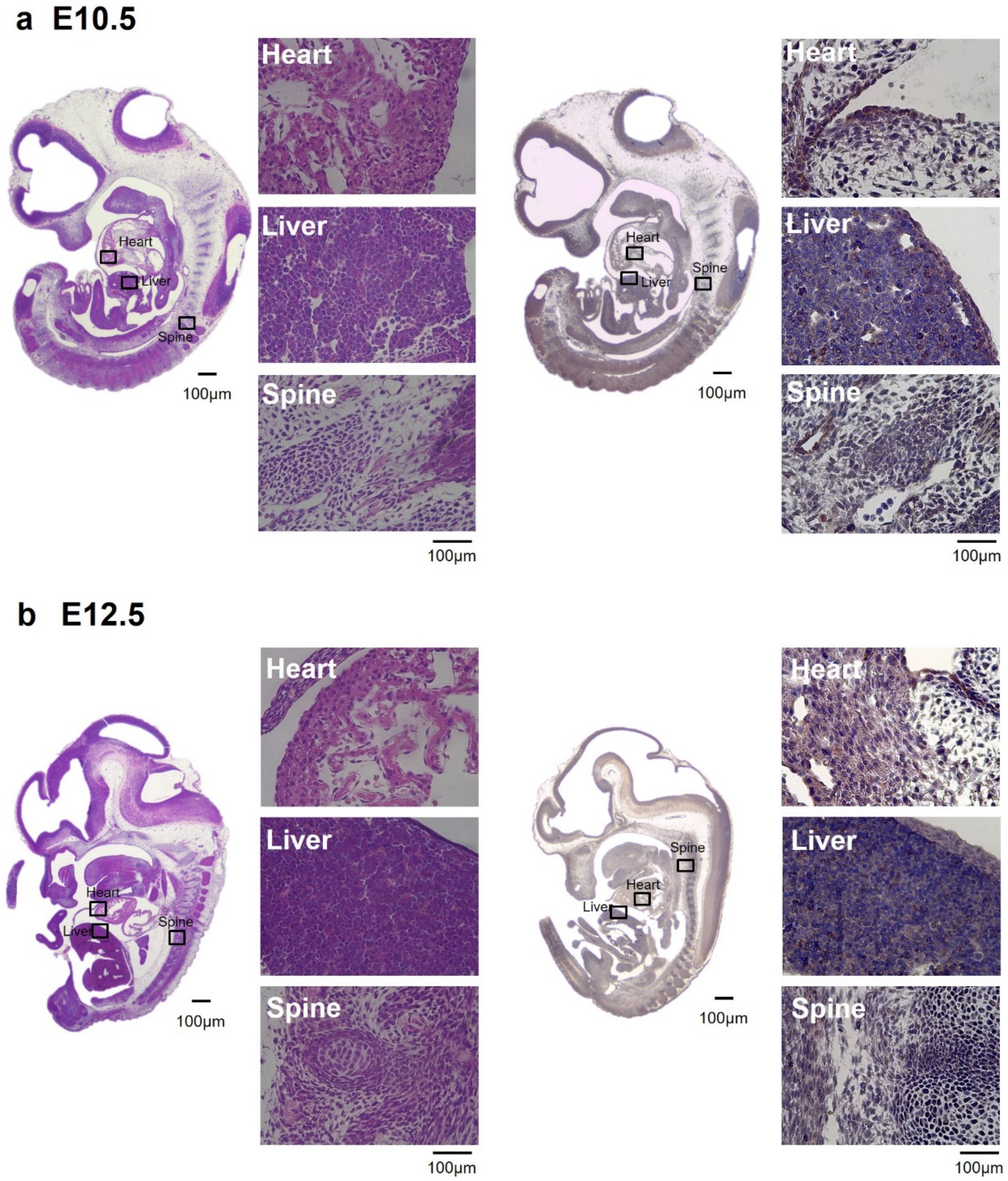

Supplementary Fig. 3. Immunohistochemistry of GFP expression in E10.5 and E12.5 embryos.

GFP expression was analyzed by immunohistochemistry (IHC) in LPAR4 lineage-tracing mice embryos at **a** E10.5 and **b** E12.5. Scale bar, 100 μm.

* LPAR4 promoter-GFP mouse

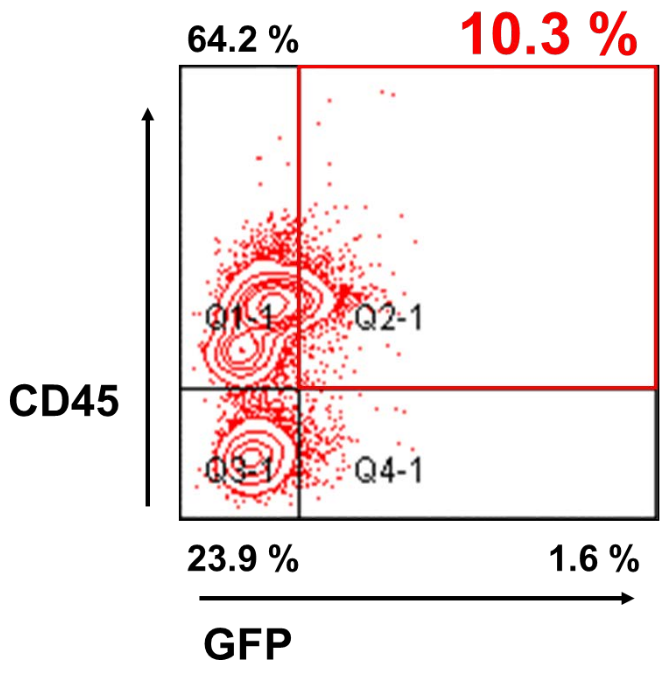

Supplementary Fig. 4. The efficiency of bone marrow transplantation (BMT).

BMT was performed by harvesting bone marrow from LPAR4 lineage-tracing mice and injecting them into wild-type mice in which BM cells were replenished through irradiation. After four weeks of BMT, FACS analysis was performed using GFP (FITC) and CD45 (PE) staining. All experiments were conducted at least in triplicate.

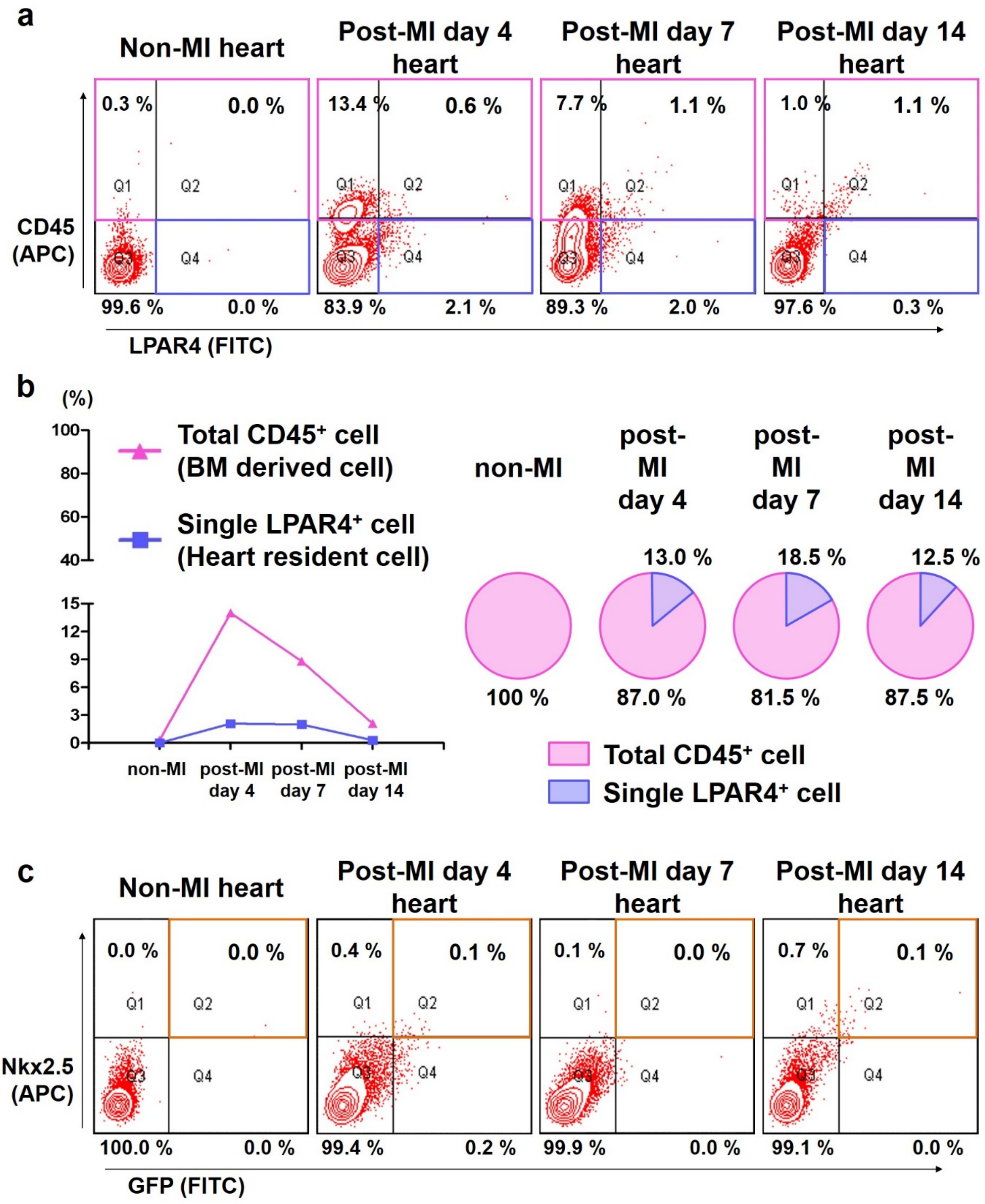

Supplementary Fig. 5. Proportion of heart-resident cells and bone marrow-derived cells in the infarcted heart after BMT.

a FACS analysis using LPAR4 (FITC) and CD45 (APC) staining in non-MI hearts after BMT and in hearts on days 4, 7, and 14 post-MI after BMT. LPAR4 represents heart-resident cells,

whereas CD45 represents BM-derived cells. **b** (Left) Line graph showing the percentage of total $CD45^+$ cells and single $LPAR4^+$ cells in non-MI and post-MI hearts. (Right) Venn diagram showing the ratio of total $CD45^+$ cells and single $LPAR4^+$ cells in non-MI and post-MI hearts. **c** FACS analysis using GFP (FITC) and Nkx2.5 (APC) staining in non-MI hearts after BMT and in hearts on days 4, 7, and 14 post-MI after BMT. None of the cells expressed either GFP or Nkx2.5. All experiments were conducted at least in triplicate.
